# A correlation study between cervical cancer and sex hormones

**DOI:** 10.3389/fonc.2024.1475052

**Published:** 2024-11-29

**Authors:** Xian Wu, Yitao You, Zhichong Guan, Yongpiao Ban, Yiping Wang, Dianhua Du, Bo Wang, Wen Wu, Yue Wen, Yixian Ren, Chunwei Wu, Xuelin Zhang, Lan Mo

**Affiliations:** ^1^ Health Management Center, The Affiliated Hospital of Guizhou Medical University, Guiyang, Guizhou, China; ^2^ School of Public Health, Guizhou Medical University, Guiyang, Guizhou, China; ^3^ Institute of Environmental and School Hygiene and Disinfection, Sichuan Center For Disease Control and Prevention, Chengdu, China; ^4^ Key Laboratory of Occupational Environment and Health, Guangzhou Twelfth People's Hospital, Guangzhou, China

**Keywords:** cervical cancer, follicle-stimulating hormone, prolactin, thin prep cytologic test, sex hormones

## Abstract

**Objective:**

The aims of this study were to investigate the association between positive TCT and sex hormone levels and to evaluate the feasibility of change in sex hormone level as a potential predictor of cervical cancer.

**Methods:**

We recruited 910 female participants from the health examination center of a hospital in Guizhou between 2019 and 2023. All participants had undergone both hematologic examinations and cervical cancer screening.

**Results:**

A total of 265 participants had positive TCT screening. Luteinizing hormone, estradiol, prolactin, and progesterone were negatively correlated with positive TCT screening. Age, systolic blood pressure, alanine aminotransferase, aspartate aminotransferase, urea, fasting blood glucose, low-density lipoprotein, total cholesterol, triglyceride, erythrocyte, follicle-stimulating hormone, and testosterone were positively correlated with positive TCT screening. Logistic regression analysis showed that prolactin, red blood cell count, and age were risk factors for cervical cancer, while FSH was a protective factor for cervical cancer. The AUC of FSH and age in the prediction model was 0.701 and 0.705, respectively.

**Conclusion:**

The incidence of cervical cancer increases with increased age and follicle poietin level. At the same time, the increase in FSH level has a certain predictive value for the incidence of cervical cancer.

## Introduction

Cervical cancer is one of the most common cancers in the world, and is also a gynecologic malignancy with a high incidence (1, 2). The peak age of cervical cancer is from 50 to 55 years. Human papilloma virus (HPV) infection, multiple sexual partners, early sexual life (<16 years of age), smoking, sexually transmitted diseases, low economic status, and immunosuppression, among other factors, result in cervical cancer (3). While the incidence of most cancers increases with age, the incidence of cervical cancer tends to stabilize at age 40–45 and may depend on the change of estrogen levels (4, 5). Women who have used oral contraceptives for more than 5 years have a higher risk of developing cervical cancer than women who have never used, and 10 years or more after stopping oral contraceptives, the risk of developing the disease is reduced. It is considered to be related to estrogen level (6). Estrogen is one of the sex hormones, and the most used sex hormone test includes six types, namely, follicle-stimulating hormone (FSH), progesterone (P), estradiol (E2), luteinizing hormone (LH), prolactin (PRL), and testosterone (T). Sex hormones promote the development of the uterus and are closely related to the replication and transmission of HPV. Estrogen and progesterone promote the expression of oncoproteins E6 and E7. Sex hormones may be a risk factor for the development of cervical cancer (7). At present, epidemiological studies focusing on the relationship between sex hormone signals such as estrogen or progesterone and cervical cancer have not been conclusive.

The routine screening programs for early cervical cancer are mainly cervical cytology, HPV test, and TCT. TCT screening greatly increased the detection rate of precancerous lesion of cervical cancer and cervical cancer. Early TCT screening detects cervical precancerous lesions. If there are inflammatory lesions or reactive hyperplasia and other abnormal changes in the early stage, they may gradually develop into malignant lesions in the later stage. Therefore, timely TCT screening is very important, and TCT is therefore widely used as an effective means of women’s “two-cancer” screening (8).

Persistent high-risk HPV infection is one of the most important epidemiological risk factors for cervical cancer, but it is not sufficient to directly or solely lead to the development of cervical disease (5). There may be other influencing factors. In addition to the response to the metabolic state of the body, the metabolic biochemical indicators in the body also play a role in balancing the body’s metabolism to a certain extent, which is related to not only the body’s immune function but also the degree of virus infection. Including alanine aminotransferase or vitamin D, such as metabolic biochemical indexes were associated with cervical cancer lesion has not been reported. This is also one of the contents of this study.

To sum up, based on the role of sex hormones in the female reproductive system and in maintaining its function, the goal of this research is to explore the possibility that sex hormone levels change as an indicator for early cervical cancer or precancerous lesions.

## Subjects and methods

The participants were recruited from the health management center of one of the Guizhou hospitals. Participants who met the following criteria were included in the study: (1) age between 20 and 65; (2) underwent TCT examination and examination of six sex hormones; (3) abnormal symptoms of cervical lesions such as erosion, ulcer, hypertrophy, hyperplasia, and contact bleeding; (4) pathological diagnosis for cervical cancer; (5) no history of surgery, such as cervical conization, and no related radiotherapy or chemotherapy; and (6) completed clinical data.

The following are the exclusion criteria: (1) pregnant or breastfeeding women; (2) taking hormone-related immune inhibitors or other known drugs that affect the sex hormone level; and (3) patients with other cancers.

**Figure 1:**
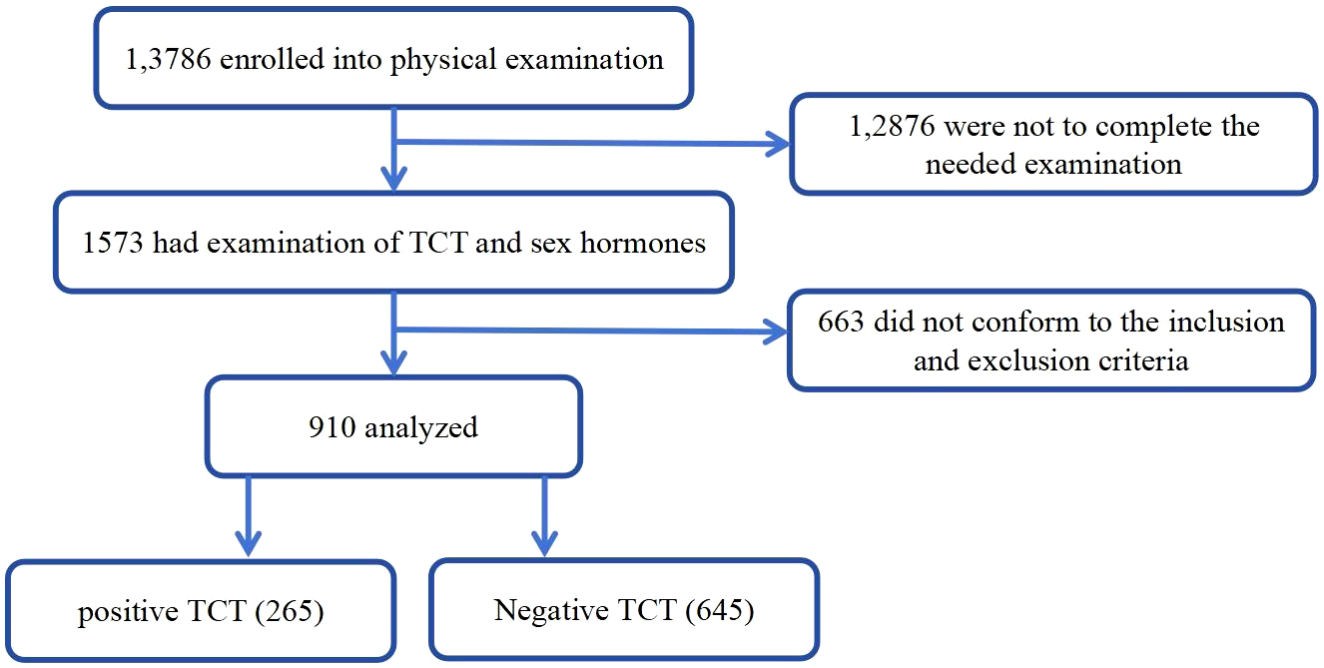
Flowchart of this study.

Enrolled participants were divided into groups with or without positive TCT test. The physical examination protocol and the consent form for this study were reviewed by the institutional review boards for both hospitals. The study flow is shown in **Figure 1**.

### Data sources

Physical examination was performed to obtain information on gender, age, blood pressure, fasting blood sugar, uric acid, and sex hormones, among others.

### The collection and testing of sex hormones in blood samples

Fasting antecubital venous blood (5 mL) from each research subject on the second to fifth day after the onset of menstruation was collected. Barcode labels with the examinee’s information were affixed to the collected blood specimen. After collection, blood specimens were sent to the laboratory promptly for testing of serum luteinizing hormone (LH), estradiol (E2), follicle-stimulating hormone (FSH), prolactin (PRL), testosterone (T), and progesterone (P). The testing process strictly adheres to the laboratory testing methods and principles of aseptic operation procedures.

### Related indicators

The specific indicators of the sex hormones included luteinizing hormone (LH), follicle-stimulating hormone (FSH), estradiol (E2), prolactin (PRL), progesterone (P), and testosterone (T). Creatinine, urea, uric acid, fasting blood glucose, alanine aminotransferase, aspartate aminotransferase, high-density lipoprotein, low-density lipoprotein, total cholesterol, triglyceride, thyroid-stimulating hormone (TSH), free triiodothyronine, free tetraiodothyronine, vitamin D3, white blood cell (urine), and red blood cell count were included.

### Definition of positive TCT

TCT test: the gross absence of intraepithelial lesions and tumor cells was regarded as normal. When the TCT test result suggested “atypical squamous cells of undetermined significance (ASC-US)”, it was judged as positive.

### Statistical analyses

All collected data were analyzed by Statistical Package for Social Science (SPSS 26.0). Continuous variables are shown as the mean ±standard deviations (SD) and categorical variables are reported as percentages (%). Differences in the characteristics of the study sample by indicator change were tested using independent analysis of *t*-tests or *χ* ^2^ tests. Correlations were analyzed by logistic regression model. *p* < 0.05 was considered statistically significant. All *p*-values were two-tailed.

## Results

The characteristics of the participants are summarized in **Table 1**. A total of 950 subjects were enrolled in this study. The mean age of the participants was 46.24 years old. The study included 265 positive TCT patients and 654 individuals without positive TCT. There were statistical differences between the two groups on all indicators except BMI, diastolic blood pressure, creatinine, uric acid, high-density lipoprotein, thyrotropin, free triiodothyronine, free tetraiodothyronine, vitamin D3, and leukocyte (urine) levels. Participants with positive TCT had a significantly higher age, systolic blood pressure, alanine aminotransferase, aspartate aminotransferase, urea, fasting blood glucose, low-density lipoprotein, total cholesterol, triglyceride, red blood cell count, follicle poietin, and luteinizing hormone than those without positive TCT, while participants without positive TCT had a lower estradiol and prolactin than those with positive TCT as presented in **Table 1**.

**Table 1:** Lab values of all participants with and without positive TCT.

Characteristics (mean±SD)	All participants (n=910)	Positive TCT (n=265)	Negative TCT (n=645)	t	*P*
Age	46.24±8.71	50.25±8.38	44.59±8.30	9.275	<0.001
BMI	23.21±3.06	23.52±2.95	23.06±3.09	1.939	0.053
SBP	116.73±15.71	118.91±16.81	115.78±15.12	2.489	0.013
DBP	71.37±10.29	72.38±10.72	70.93±10.07	1.793	0.074
ALT	18.80±11.80	20.33±13.45	18.16±10.96	2.492	0.013
AST	20.74±7.90	22.31±9.72	20.08±6.89	3.830	<0.001
Cr	57.03±8.80	57.78±9.56	56.73±8.462	1.451	0.148
UR	4.64±1.18	4.88±1.23	4.55±1.14	3.591	<0.001
UA	293.89±63.23	294.39±61.42	293.70±63.96	0.142	0.887
FFBG	5.00±0.67	5.11±0.90	4.96±0.55	2.212	0.028
HDL	1.49±0.37	1.50±0.37	1.49±0.37	0.444	0.657
LDL	3.02±0.76	3.13±0.78	2.98±0.75	2.710	0.007
TC	4.92±0.87	5.09±0.87	4.84±0.86	3.892	<0.001
TG	1.43±0.95	1.55±0.95	1.37±0.94	2.536	0.012
TSH	3.22±3.66	3.50±6.11	3.11±1.78	1.309	0.191
FFT3	4.70±1.27	4.77±2.05	4.66±0.70	0.784	0.434
FT4	16.51±3.22	16.53±4.00	16.50±2.83	0.104	0.917
VD3	29.91±9.26	30.19±9.37	29.75±9.22	0.466	0.641
WBC	59.71±886.98	27.01±774.92	71.01±1028.09	0.095	0.366
RBC	4.64±0.38	4.69±0.41	4.63±0.37	2.130	0.034
FSH	37.92±35.19	55.09±34.33	30.86±33.07	9.772	<0.001
LH	24.85±19.48	32.01±18.72	21.90±19.02	7.367	<0.001
E2	270.81±430.58	140.51±251.94	324.35±475.19	5.962	<0.001
PRL	328.33±246.83	268.30±153.63	353.00±272.44	4.759	<0.001
P	4.05±12.13	2.41±9.98	4.72±12.86	2.614	0.009
T	0.63±0.42	0.52±0.37	0.68±0.43	5.358	<0.001

**Table 2** illustrates the relationship between positive TCT and the aforementioned indicators. Luteinizing hormone, estradiol, prolactin, and progesterone were negatively correlated with positive TCT screening; that is, the probability of positive TCT screening decreased with the increase of luteinizing hormone, estradiol, prolactin and progesterone levels. Age, systolic blood pressure, alanine aminotransferase, aspartate aminotransferase, urea, fasting blood glucose, low-density lipoprotein, total cholesterol, triglyceride, erythrocyte, Follicle-Stimulating Hormone, and testosterone were positively correlated with positive TCT screening; that is, the probability of positive TCT screening increased with the increase of these levels.

**Table 2:** Correlation of positive TCT with lab value.

Characteristics	TCT	
	*r*	*P*
Age	0.295**	<0.001
SBP	0.092**	0.010
ALT	0.084*	0.013
AST	0.129**	<0.001
UR	0.129**	<0.001
FBG	0.095**	0.006
LDH	0.134**	<0.001
TC	0.088*	0.011
TG	0.075*	0.027
RBC	0.313**	<0.001
FSH	0.236**	<0.001
LH	-0.194**	<0.001
E2	-0.156**	<0.001
PRL	-0.086**	0.009
P	-0.175**	<0.001
T	0.295**	<0.001

**At 0.01 level (two-tailed), correlation is obvious.

* At 0.05 level (two-tailed), correlation is obvious.

Logistic regression analysis was used to estimate the odds ratio (OR) and 95% confidence interval (CI) for the prevalence of positive TCT in the population, as shown in **Table 3**. Follicle-producing hormone, prolactin, age, and red blood cell count were associated with the prevalence of positive TCT. It is found that follicle-producing hormone, red blood cell counts, and age were used as reference, and the risk of positive TCT gradually increased with rising follicle-producing hormone (OR = 1.022, 95% CI: 1.011–1.034), red blood cell count (OR = 1.809, 95% CI: 1.176–2.784), and age (OR = 1.080, 95% CI: 1.054–1.108). Increased level of prolactin (OR = 0.999, 95% CI: 0.998–1.000) was a protective factor for positive TCT.

**Table 3:** Correlation of positive TCT with lab value.

Variables	β	S_X_	Waldχ^2^	OR	95%CI	P
FSH	0.022	0.006	15.858	0.978	0.967-0.989	<0.001
LH	-0.016	0.009	3.099	1.016	0.998-1.035	0.078
E2	-0.001	0	2.963	1.001	1-1.001	0.085
PRL	-0.001	0	4.49	1.001	1-1.002	0.034
P	0.002	0.008	0.051	0.998	0.983-1.014	0.821
T	-0.339	0.217	2.437	1.404	0.917-2.149	0.119
Age	0.077	0.013	36.847	1.080	1.054-1.108	<0.001
ALT	0.003	0.012	0.071	1.003	0.98-1.027	0.79
AST	0.019	0.018	1.101	1.019	0.984-1.056	0.294
UA	0.087	0.074	1.408	1.091	0.945-1.26	0.235
LDL	0.144	0.113	1.637	1.155	0.926-1.26	0.201
RBC	0.593	0.22	7.265	1.809	1.176-2.784	0.007

**Figure 2:**
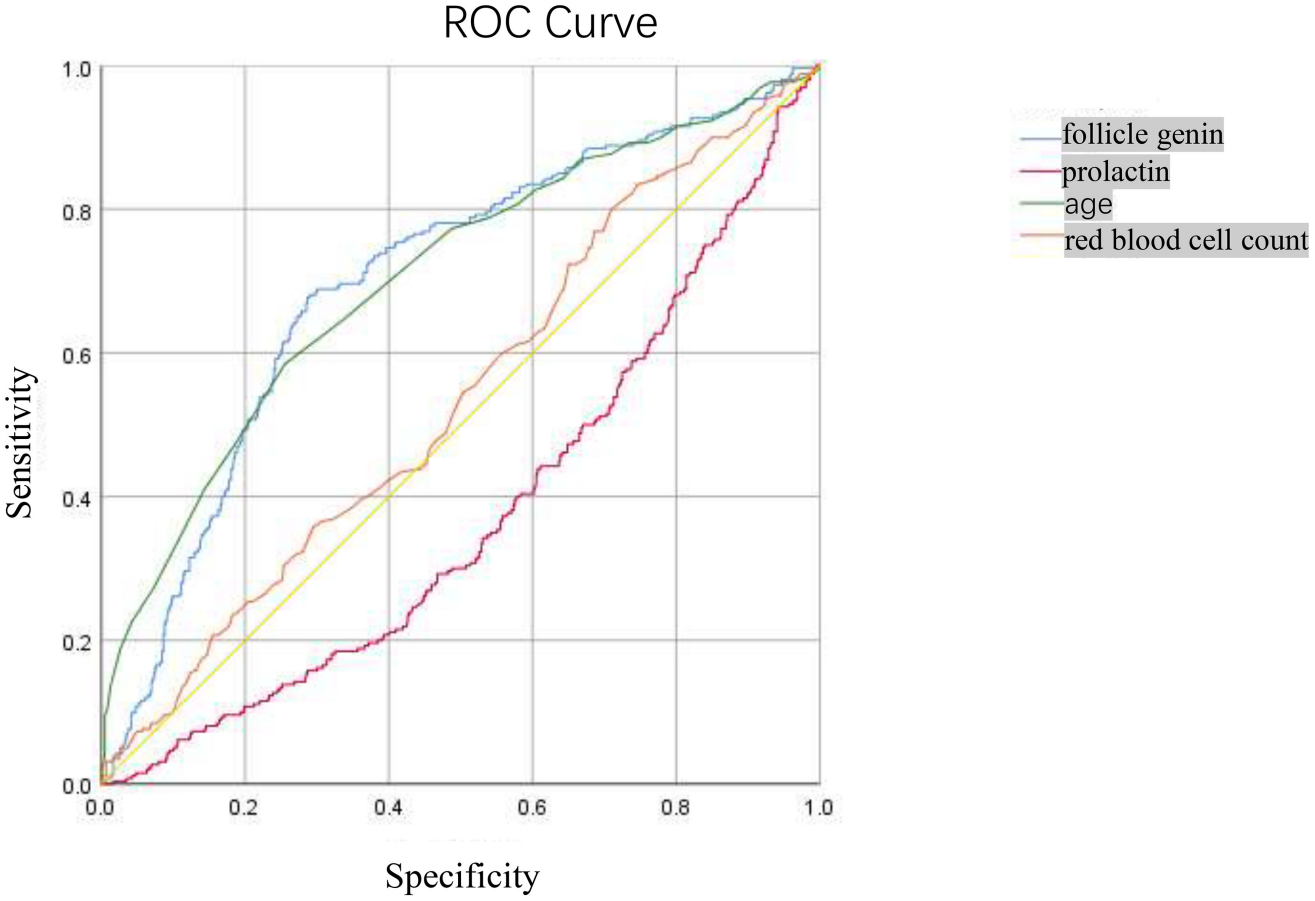
The ROC Curve for predicting the correlation between positive TCT screening and follicle-stimulating hormone, prolactin.

ROC curves were plotted to evaluate the efficiency of Follicle-Stimulating Hormone, prolactin, age, and red blood cell count in predicting the outcome of positive TCT in patients, as shown in **Figure 2**. We observed that the area under the curve (AUC) of Follicle-Stimulating Hormone, prolactin, age, and red blood cell count was 0.701 (95% CI, 0.663–0.739), 0.02 (95% CI, 0.340–0.419), 0.705 (95% CI, 0.666–0.744), and 0.021 (95% CI, 0.495–0.578), respectively. The optimal diagnostic cutoff point of Follicle-Stimulating Hormone was 44.625, the sensitivity was 67.2%, and the specificity was 71.6%. The optimal diagnostic cutoff point of age was 50.50, the sensitivity was 58.5%, and the specificity was 74.5%.

## Discussion

This is a study that aims to investigate the relationship between TCT screening and sex hormones in female patients from the health management center of a top-tier hospital from January 2019 to September 2023. We found that follicle-stimulating hormone may be a potential cervical cancer prediction indicator, and this provided a theoretical basis to better strengthen the prevention of cervical cancer in this region.

In this study, the average age of the positive group (50.25 ± 8.38 years) was higher than that of the negative group (44.59 ± 8.30 years). It indicated that within a certain age range, the risk of cervical cancer increases with rising age, which may be related to the changes of hormone levels and organ function. Compared with the negative group, the levels of systolic blood pressure, alanine aminotransferase, aspartate aminotransferase, urea, fasting blood glucose, low-density lipoprotein, total cholesterol, triglyceride, red blood cell count, follicle-stimulating hormone, luteinizing hormone, estradiol, prolactin, progesterone, and testosterone were higher. Pearson correlation analysis was used to resolve the above indicators and TCT positive correlation, and the results showed that luteinizing hormone, estradiol, prolactin, and progesterone were negatively correlated with positive TCT screening. With the luteinizing hormone, estradiol, prolactin, and progesterone levels rising, the possibility of positive TCT declined. Age, systolic blood pressure, alanine aminotransferase, aspartate aminotransferase, urea, fasting blood glucose, low-density lipoprotein, total cholesterol, triglyceride, red blood cells, follicle-stimulating hormone, and testosterone were positively correlated with the positive rate of TCT screening, and the positive rate of TCT increased with the increase of these indicators. Logistic regression analysis results show that prolactin, red blood cell count, and age were risk factors for positive TCT. Follicle-stimulating hormone is a protective factor for positive TCT; follicular hormone increases 1 unit of TCT screening positive for an increased risk of 1.022 times. Every increase of 1 unit of prolactin had a 0.1% lower risk of TCT screening positive. For each unit increase in red blood cell count, the risk of positive TCT screening increases by 1.80 times. TCT screening positive for an increased risk of 1.08 times when age increased 1 years. Furthermore, an ROC curve was used to examine the prediction effect of age, prolactin, red blood cell counts, and follicle-stimulating hormone for positive TCT. The results found that follicular hormone (AUC = 0.701) and age (AUC = 0.705) had predictive values for TCT screening positive.

Previous studies have found that age is also associated with a high HPV infection rate, and there are two peaks of HPV infection in younger and older women (5). It is shown that age was associated with cervical lesions, and age was likely to be an independent risk factor that affects cervical lesion progress (9). In this study, our results show that age was a risk factor for cervical cancer; as age increases, the risk of cervical cancer became higher. The age of the diseased group is 50.25 ± 8.38 years, which prompted the idea that perimenopausal and postmenopausal women need more attention in terms of gynecological disease screening.

It is easy to be ignored that some metabolic biochemical indicators such as blood pressure, ALT, or other hormones effect the happening of cervical cancer. In this research, we found that levels of systolic, glutamic-pyruvic transaminase, aspartate aminotransferase, urea, fasting blood sugar, low-density lipoprotein cholesterol (LDL-C), total cholesterol, triglycerides, and the level of red blood cell count of the patients with positive TCT were slightly higher than the TCT-negative patients, suggesting that in the former, the patient’s metabolic biochemical indicators’ subtle changes have occurred. These changes may cause abnormal changes in cervical cells. In recent years, scholars have reported the expression of red blood cell distribution width (RDW) in various common tumors such as breast cancer, colorectal cancer, and bladder cancer and its role in diagnosis or prognosis (10–12). The results of our analysis showed that the risk of positive TCT screening increased by 1.8 times for every 1 unit increase in red blood cell count. The change had a certain predictive value for cervical abnormal lesions, indicating that the increase in red blood cell count would also promote cervical abnormal changes.

In addition to the changes in metabolic biochemical indicators, this research is more focused on the influence of sex hormones on changes in cervical lesions. Because sex hormones are hormones that promote the development of the female uterus, cervical epithelial cells will secrete mucus in response to changes in estrogen and progesterone levels. Estrogen and progesterone also promote the expression of cancer proteins E6 and E7. Besides female progesterone, the hormones also include other indicators: six kinds of sex hormones commonly used in the clinic to analyze the female endocrine condition and for clinical evaluation. A comprehensive analysis of sex hormone levels is needed to determine whether some lesions appear in the reproductive system (7). Follicle-stimulating hormone and luteinizing hormone generated from the pituitary gland promote the secretion of estrogen. Prolactin accelerates gonad development and prompts corpus luteum-generated hormone receptors. The main role of progesterone is to improve pregnancy. Estradiol maintains the function of the body’s accessory organs and has a synergistic effect with progesterone (13).

Previous studies have confirmed that the change in estrogen levels is related to the incidence of cervical cancer, while there are only a few reports that associate commonly used sex hormones, including follicle-stimulating hormone, luteinizing hormone, prolactin, progesterone, and testosterone, with the incidence of cervical cancer (14). Our study found that follicular hormone is a risk factor for cervical cancer; an increase in follicular hormone levels is associated with an increased risk of cervical cancer. Follicular hormones could be a predictor for the occurrence of cervical cancer. However, the levels of luteinizing hormone, progesterone, and testosterone in TCT-positive patients were higher than those in the normal group, but the effect of these three hormones on the occurrence of cervical cancer has not been found.

## Conclusion

Abnormal sex hormones and aging are closely related to the occurrence of cervical cancer. However, the correlation between subsequent changes in sex hormones and the prognosis of cervical cancer is not clear. Our study confirms that the detection of sex hormones may become a new target for the prevention of cervical cancer. Therefore, it is vital to increase the sample size of a study to establish a multi-center and prospective research. Further studies are needed to follow-up on the prognosis of patients and determine experimental indicators.

## Data Availability

The raw data supporting the conclusions of this article will be made available by the authors, without undue reservation.

## References

[1] Siegel RL Giaquinto AN Jemal A. Cancer statistics, 2024. CA: A Cancer J Clin. (2024) 74:12–49. doi: 10.3322/caac.21820 38230766

[2] Bouvard V Wentzensen N Mackie A Berkhof J Brotherton J Giorgi-Rossi P. The IARC perspective on cervical cancer screening. N Engl J Med. (2021) 385(20):1908-18. doi: 10.1056/NEJMsr2030640 PMC12125667 34758259

[3] Yang D Zhang J Cui X Ma J Wang C Piao H. Risk factors associated with human papillomavirus infection, cervical cancer, and precancerous lesions in large-scale population screening. Front Microbiol. (2022) 13. doi: 10.3389/fmicb.2022.914516 PMC9282163 35847094

[4] Fontham ETH Wolf AMD Church TR Etzioni R Flowers CR Herzig A. Cervical cancer screening for individuals at average risk: 2020 guideline update from the American Cancer Society. CA: A Cancer J Clin. (2020) 70:321–46. doi: 10.3322/caac.21628 32729638

[5] Wong JPH Vahabi M Miholjcic J Tan V Owino M Li ATW. Knowledge of HPV/cervical cancer and acceptability of HPV self-sampling among women living with HIV: A scoping review. Curr Oncol. (2018) 25:73–82. doi: 10.3747/co.25.3855 29507498 PMC5832294

[6] Asthana S Busa V Labani S. Oral contraceptives use and risk of cervical cancer—A systematic review & meta-analysis. Eur J Obstetrics Gynecology Reprod Biol. (2020) 247:163–75. doi: 10.1016/j.ejogrb.2020.02.014 32114321

[7] Lee S-A Baik S Chung S-H. Functional roles of female sex hormones and their nuclear receptors in cervical cancer. Essays Biochem. (2021) 65:941–50. doi: 10.1042/EBC20200175 PMC8935983 34156060

[8] Yang Y Xu L Yuan S Lv J Chen P Wang W. Optimal screening and detection strategies for cervical lesions: A retrospective study. J Cancer. (2024) 15:3612–24. doi: 10.7150/jca.96128 PMC11134435 38817879

[9] Bruni L Albero G Rowley J Alemany L Arbyn M Giuliano AR. Global and regional estimates of genital human papillomavirus prevalence among men: a systematic review and meta-analysis. Lancet Global Health. (2023) 11:e1345–62. doi: 10.1016/S2214-109X(23)00305-4 PMC10447222 37591583

[10] Ma W Mao S Bao M Wu Y Guo Y Liu J. Prognostic significance of red cell distribution width in bladder cancer. Trans Andrology Urol. (2020) 9:295–302. doi: 10.21037/tau.2020.03.08 PMC7215002 32420135

[11] Yao D Wang Z Cai H Li Y Li B. Relationship between red cell distribution width and prognosis in patients with breast cancer after operation: a retrospective cohort study. Bioscience Rep. (2019) 39:1-9. doi: 10.1042/BSR20190740 PMC6629944 31262969

[12] Shi C Xie M Li L Li K Hu B-L. The association and diagnostic value of red blood cell distribution width in colorectal cancer. Medicine. (2019) 98:1-6. doi: 10.1097/MD.0000000000015560 PMC6531168 31083220

[13] Ciaffi J van Leeuwen NM Schoones JW Huizinga TWJ de Vries-Bouwstra JK. Sex hormones and sex hormone-targeting therapies in systemic sclerosis: A systematic literature review. Semin Arthritis Rheumatism. (2020) 50:140–8. doi: 10.1016/j.semarthrit.2019.07.007 31362894

[14] Chung S-H Franceschi S Lambert PF. Estrogen and ERα: Culprits in cervical cancer? Trends Endocrinol Metab. (2010) 21:504–11. doi: 10.1016/j.tem.2010.03.005 PMC2914219 20456973

